# Simulated Cholinergic Reinnervation of *β* (INS-1) Cells: Antidiabetic Utility of Heterotypic Pseudoislets Containing *β* Cell and Cholinergic Cell

**DOI:** 10.1155/2018/1505307

**Published:** 2018-03-20

**Authors:** Ao Jiao, Feng Li, Chengshuo Zhang, Wu Lv, Baomin Chen, Jialin Zhang

**Affiliations:** Hepatobiliary Surgery Department and Unit of Organ Transplantation, The First Hospital of China Medical University, Shenyang 110001, China

## Abstract

Cholinergic neurons can functionally support pancreatic islets in controlling blood sugar levels. However, in islet transplantation, the level of cholinergic reinnervation is significantly lower compared to orthotopic pancreatic islets. This abnormal reinnervation affects the survival and function of islet grafts. In this study, the cholinergic reinnervation of beta cells was simulated by 2D and 3D coculture of INS-1 and NG108-15 cells. In 2D culture conditions, 20 mM glucose induced a 1.24-fold increase (*p* < 0.0001) in insulin secretion from the coculture group, while in the 3D culture condition, a 1.78-fold increase (*p* < 0.0001) in insulin secretion from heterotypic pseudoislet group was observed. Glucose-stimulated insulin secretion (GSIS) from 2D INS-1 cells showed minimal changes when compared to 3D structures. E-cadherin expressed in INS-1 and NG108-15 cells was the key adhesion molecule for the formation of heterotypic pseudoislets. NG108-15 cells hardly affected the proliferation of INS-1 cells in vitro. Heterotypic pseudoislet transplantation recipient mice reverted to normoglycemic levels faster and had a greater blood glucose clearance compared to INS-1 pseudoislet recipient mice. In conclusion, cholinergic cells can promote insulin-secreting cells to function better in vitro and in vivo and E-cadherin plays an important role in the formation of heterotypic pseudoislets.

## 1. Introduction

Islet transplantation is a beneficial approach for the treatment of type 1 diabetes (T1DM). However, patients with poor glycemic control, poor graft implantation and survival, and the shortage of organs for transplantation remain significant barriers to this therapy [1–3]. In addition, immune rejection, revascularization, and poor reinnervation are significant obstacles for the survival and function of islet grafts [4, 5].

Under physiological conditions, insulin secretion is controlled by the sympathetic and parasympathetic nervous systems. When the sympathetic nerve is stimulated, it releases norepinephrine, which inhibits insulin secretion by inducing vasoconstriction and suppressing *β* cell function [6]. In contrast, when the parasympathetic nerve is stimulated, it can promote insulin secretion by releasing acetylcholine, which activates muscarinic receptors in *β* cells [7]. In animal islet cell transplantation models, the density of the grafts' cholinergic innervation when implanted in the liver, spleen, or renal capsule was significantly lower than those islets in situ; however, there were no significant differences in the density of the grafts' adrenergic innervations [8]. Consequently, we investigated whether cholinergic cells can improve the function of *β* cells in vitro and in vivo.

INS-1 cells are widely used as rat islet *β* cell models for diabetes research. They express M1 and M3 receptors, which are activated by carbachol to promote insulin release [9]. INS-1 cells can also form pseudoislets (PIs) in three-dimensional (3D) culture condition due to the expression of adhesion molecules like E-cadherin [10]. The NG108-15 cell line has the ability to release acetylcholine and was created by fusing mouse N18TG2 neuroblastoma cells with rat C6-BU-1 glioma cells in the presence of inactivated Sendai virus [11]. This cell line is frequently used as a cholinergic cell line to explore neuronal functions [12]. NG108-15 cells also have the ability to form spheroidal structure via 3D coculture with the help of relevant supporting cell lines [13].

**Figure 1:**
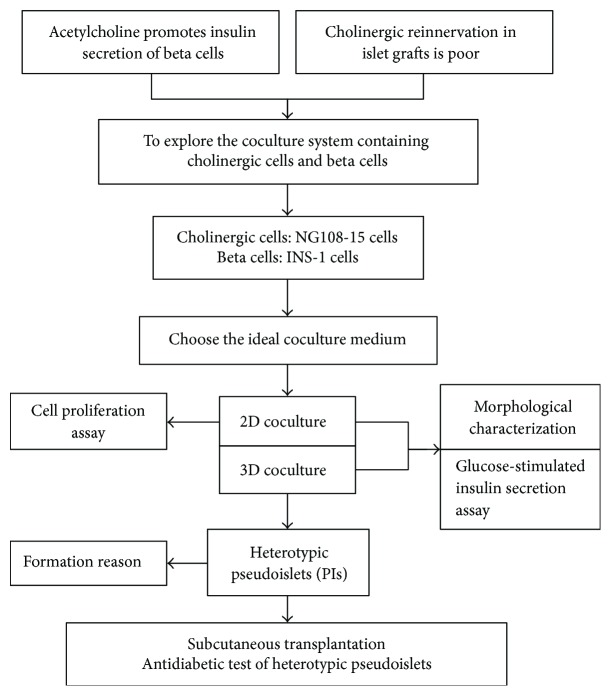
A flowchart of the study design.

In this study, therapeutic potential of heterotypic pseudoislets generated from INS-1 *β* cells and the cholinergic cell line NG108-15 was examined. Specifically, this involved comparing the function of heterotypic INS-1 and NG108-15 pseudoislets and homotypic INS-1 pseudoislets in vitro. The comparison of the antidiabetic effects of both types of pseudoislets was performed in vivo by subcutaneous transplantation into streptozotocin-induced diabetic BALB/c nu/nu mice.  Figure 1 shows the experimental design of this study.

## 2. Materials and Methods

### 2.1. Cell Culture

INS-1 cells (obtained from Bioleaf Biotech, Shanghai, China) were derived from a rat insulinoma. Cells were cultured in RPMI 1640 (GIBCO, California, USA) supplemented with 10% (*v*/*v*) fetal bovine serum (FBS) (10099141, GIBCO, Australia), 50 *μ*M *β*-mercaptoethanol (Solarbio, Beijing, China), 10 mM HEPES (Solarbio), 2 mM L-glutamine (Solarbio), 1 mM Na pyruvate (Solarbio), 100 U/mL penicillin (Solarbio), and 100 *μ*g/mL streptomycin (Solarbio), as described previously [14]. NG108-15 cells (obtained from China Center for Type Culture Collection (CCTCC), Wuhan, China) were cultured in Dulbecco's modified essential media (DMEM) (GIBCO) supplemented with 10% (*v*/*v*) FBS (GIBCO) and 1 × HAT (100 *μ*M hypoxanthine, 0.4 *μ*M aminopterin, and 16 *μ*M thymidine) (H0262, Sigma). The cells were maintained at 37°C in a humidified atmosphere containing 95% air and 5% CO_2_.

### 2.2. Animals

Female BALB/c nu/nu mice (7 weeks) were obtained from HFK Bioscience (Beijing, China). Subcutaneous pseudoislet implantation was performed under isoflurane (RWD, Shenzhen, China) inhalation anesthesia. All animal experiments were conducted in accordance with the National Institute of Health Guide and Use of Laboratory Animals and were approved by China Medical University Animal Care and Use Committee.

### 2.3. Establishment of Stable Cell Lines Expressing Red and Green Fluorescent Protein

In order to visualize the 2D and 3D coculture system, we used lentivirus expressing either red (RFP) or green fluorescent protein (GFP) to establish INS-1 and NG108-15 stable cell lines. Lentivirus was obtained from Genechem (Shanghai, China). INS-1 and NG108-15 cells were seeded at a density of 2 × 10^5^ cells per well in 6-well plates (Corning, NY, USA) and maintained at 37°C with 5% CO_2_ for 24 hours. INS-1 cells were then infected with GFP lentivirus (MOI = 4), and NG108-15 cells were infected with RFP lentivirus (MOI = 4). Puromycin (2 *μ*g/ml) (Solarbio) was used to select for transfected cells. One week later, puromycin-resistant colonies were selected, and colonies were expanded to generate stable cell lines in the presence of 2 *μ*g/ml puromycin.

### 2.4. 2D and 3D Coculture Model

INS-1 medium supplemented with 1 × HT (100 *μ*M hypoxanthine, 16 *μ*M thymidine) (H0137, Sigma) was used as the coculture medium. For 2D coculture, a 1 : 1 combination of INS-1 and NG108-15 cells was seeded at a density of 8 × 10^4^ cells per well into 12-well flat-bottomed tissue culture plates (Corning) for 72 hours (media was replaced after 48 hours). For 3D coculture, a 1 : 1 combination of INS-1 and NG108-15 cells was seeded at a density of 4 × 10^5^ cells per well into 6-well ultra-low attachment, flat-bottomed tissue culture plates (Corning) for 72 hours (media was replaced after 48 hours).

### 2.5. Measurement of Cell Viability

**Figure 2:**
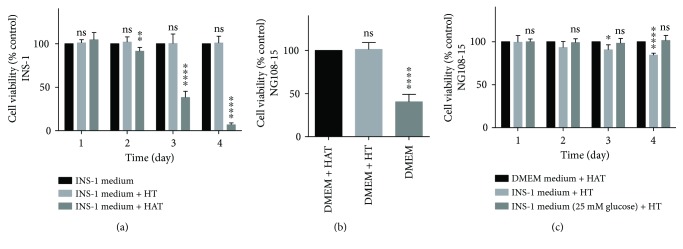
INS-1 medium supplemented with HT is the ideal medium for short-term coculture. (a, b, and c) Cellular viability was determined by CCK-8 kits and the data were expressed as percentages of untreated control cells. Results are means ± SD of four independent experiments. (a) INS-1 cells were cultured in INS-1 medium (control) with or without HT or HAT. ^∗∗^ *p* < 0.01 and ^∗∗∗∗^ *p* < 0.0001 and ns versus INS-1 medium group, *n* = 4. (b) NG108-15 cells were cultured in DMEM medium with or without HT or HAT. Cellular viability of NG108-15 cells was determined after 24-hour culture. ^∗∗∗∗^ *p* < 0.0001 and ns versus DMEM + HAT group, *n* = 4. (c) NG108-15 cells were cultured in DMEM medium with HAT, INS-1 medium with HT, and INS-1 medium (25 mM Glucose) with HT, respectively. ^∗^ *p* < 0.05 and ^∗∗∗∗^ *p* < 0.0001 and ns versus DMEM medium + HAT group, *n* = 4.

**Figure 3:**
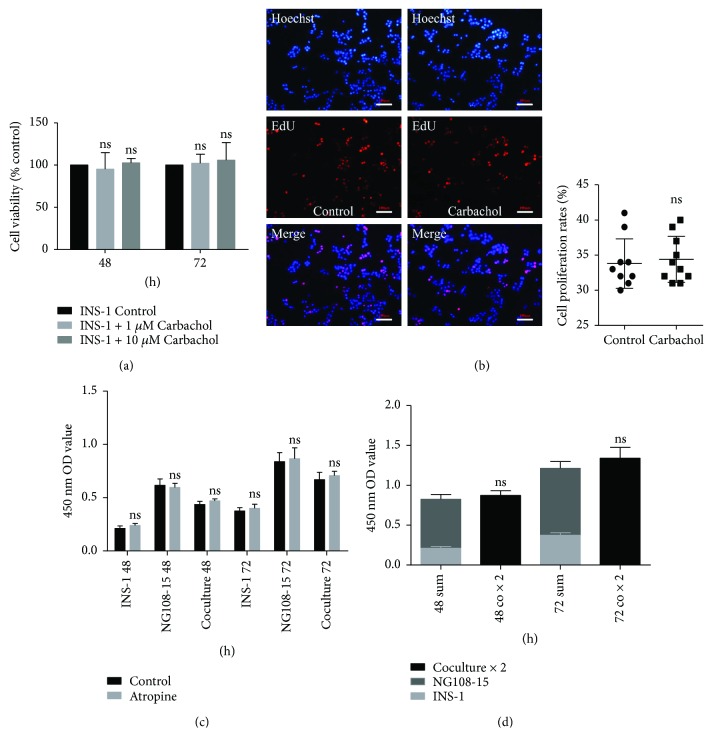
Coculture system hardly affects proliferation of INS-1 cells. (a, c, and d) Cellular viability was determined by CCK-8 kits. Results are means ± SD of four independent experiments. (a) INS-1 cells were cultured in complete medium (control) with or without carbachol (1 *μ*M, 10 *μ*M) for 48 and 72 hours. The data were expressed as percentages of untreated control cells and ns versus INS-1 control group, *n* = 4. (b) INS-1 cells were cultured in complete medium (control) with or without 10 *μ*M carbachol for 72 hours. EdU-labeled INS-1 cells were assessed using fluorescent microscopy. All cell nuclei were Hoechst 33342-positive (blue), and all replicating cells were EdU-positive (red). The scatter plot represents the percentage of EdU-labeled proliferative INS-1 cells. Data were presented as the mean ± SD. ns versus control group, *n* = 10. (c) Cells were cultured in coculture medium (control) with or without 10 *μ*M atropine sulfate for 48 and 72 hours. The data were expressed as 450 nm OD value and ns versus control group, *n* = 4. (d) Cells were cultured in coculture medium for 48 and 72 hours. The data were expressed as 450 nm OD value and ns versus the sum of INS-1 group and NG108-15 group, *n* = 4.

Cell viability was determined using CCK-8 kits (Beyotime, Shanghai, China). For finding the most ideal coculture medium, cells were seeded in 96-well plates (Corning) at a concentration of 5 × 10^3^ cells per well, allowed to adhere overnight, and subsequently exposed to different complete media (100 *μ*l) including INS-1 medium supplemented with or without HT or HAT and DMEM medium with or without HT or HAT. For assessing the proliferation of coculture system, a 1 : 1 combination of INS-1 and NG108-15 cells was seeded in 96-well plates at a density of 5 × 10^3^ cells per well, allowed to adhere overnight, and subsequently exposed to different coculture media (100 *μ*l) with or without atropine sulfate (Meilun, Dalian, China) or carbachol (Meilun). Control group cells were also seeded at the same concentration. After a certain period of culture (Figures 2 and 3), 10 *μ*l of CCK-8 was added to each well and the cells were incubated for 1 h. The absorbance at 450 nm was measured using a microplate reader. The viability ratio was calculated according to the following formula: Viability ratio = [(absorbance of experimental group − absorbance of blank group)/(absorbance of control group − absorbance of blank group)] × 100%.

### 2.6. Assessment of Insulin Release and Total Insulin Content

INS-1 cells were cultured using the same coculture conditions mentioned previously. After 18 hours of incubation with 5.6 mM glucose medium (RPMI 1640 without glucose (R1383, Sigma) + glucose (Solarbio) + coculture components mentioned above), secretion assays were performed. Adherent cells and pseudoislets were first preincubated for 60 min at 37°C in Krebs-Ringer bicarbonate HEPES buffer (KRBH) (115 mM NaCl, 4.7 mM KCl, 1.28 mM CaCl_2_, 1.2 mM MgSO_4_, 10 mM NaHCO_3_, and 20 mM HEPES) containing 1.1 mmol/l glucose supplemented with 1 mg/ml bovine serum albumin (BSA) (Solarbio) [15]. Following preincubation, they were then exposed to 1.1 mM or 20 mM glucose with or without 10 *μ*M carbachol (Meilun) or 10 *μ*M atropine sulfate (Meilun) for 60 min. The supernatants were then collected from each well and secreted insulin was determined using the Ultrasensitive Rat Insulin ELISA kit (Mercodia, Sweden) according to the manufacturer's protocol. Insulin secretion data were normalized to total insulin content of the cells collected from each well using RIPA buffer (Beyotime). Total insulin content also was determined by ELISA (Mercodia) [16].

### 2.7. Assessment of E-Cadherin Expression by Western Blot

Total cell lysates were analyzed by Western blot as described previously [14]. Briefly, total protein from cells was extracted using RIPA buffer (Beyotime) and protein concentrations were determined using the BCA kit (Beyotime). Twenty micrograms of total protein extracts were resolved by 10% SDS-PAGE and then subsequently electroblotted onto PVDF membranes. Blots were blocked with 5% nonfat milk (Boster Biological Technology, Wuhan, China) for 30 min and then probed with 1 : 1000-diluted primary *β*-actin (60008-1-Ig, Proteintech) or E-cadherin (20874-1-AP, Proteintech) antibodies and incubated overnight at 4°C, followed by horseradish peroxidase-conjugated secondary antibodies for 1.5 h at room temperature. Proteins were visualized using ECL reagent (Beyotime). The results were scanned using the Bio-Rad Gel Doc XR+ System, and densitometric analysis of the scanned images was performed using the Image Lab 5.0 software.

### 2.8. Assessment of E-Cadherin Expression by Immunofluorescence

Cell climbing slices and heterotypic pseudoislets were fixed in 2% paraformaldehyde (*w*/*v*) (Servicebio, Wuhan, China) for 10 min. They were then rinsed in TBSTx buffer (Beyotime) and subsequently blocked for 60 min with TBSTx buffer supplemented with 5% BSA (*w*/*v*) (Solarbio). They were then incubated with 1/100 dilution of anti-E-cadherin antibody (ab76055, Abcam) in TBSTx buffer supplemented with 5% BSA (*w*/*v*) (Solarbio) overnight at 4°C. They were then washed in TBSTx buffer and reincubated at room temperature for 1 hour with Alexa Fluor 350-conjugated secondary antibody (Beyotime). Finally, they were washed three times in TBSTx buffer and imaged using an inverted fluorescence microscope [10].

### 2.9. Impact of Anti-E-Cadherin Antibody on Heterotypic Pseudoislet Formation

A 1 : 1 ratio of INS-1-GFP and NG108-15-RFP cells was seeded at a density of 2 × 10^4^ cells per well into 96-well ultra-low attachment, flat-bottomed tissue culture plates (Corning) and maintained in coculture medium containing 1/100 dilution of E-cadherin antibody (ab76055, Abcam) or 1/100 dilution of *β*-actin antibody (60008-1-Ig, Proteintech) for 24 hours. The number of heterotypic pseudoislets was measured using an inverted fluorescence microscope [17]. The control group was maintained in coculture medium only.

### 2.10. 5-Ethynyl-2′-Deoxyuridine (EdU) Incorporation Assay

EdU, a thymidine analog in which a terminal alkyne group substitutes the methyl group in the 5th position, is incorporated into cellular DNA during DNA replication in proliferating cells [18]. Proliferating cells were stained with EdU using the Cell-Light EdU DNA Cell Proliferation Kit (RiboBio Co., Guangzhou, China). Briefly, cells were seeded in 96-well plates at a concentration of 5 × 10^3^ cells/well, allowed to adhere overnight, and subsequently exposed to different complete media (100 *μ*l) with or without carbachol (10 *μ*M). After 72 h, 50 *μ*mol/L of EdU was added to cells for 4 h at 37°C. After fixation with 4% (*w*/*v*) paraformaldehyde for 30 min, the cells were treated with 0.5% (*v*/*v*) Triton X-100 (Sigma) for 20 min and rinsed with PBS three times. Thereafter, the cells were exposed to 100 *μ*L of 1 × Apollo® reaction cocktail for 30 min and incubated with 5 *μ*g/mL of Hoechst 33342 (Solarbio) to stain the cell nuclei for 30 min. EdU-labeled cells and Hoechst 33342-stained cells were counted in 10 random fields of view using a fluorescent microscope. The percentage of EdU-positive cells was calculated as the number of EdU-positive cells/the number of Hoechst-positive cells.

### 2.11. Diabetes Induction and Pseudoislet Transplantation

One week before transplantation, female BALB/c nu/nu mice (8–10 weeks) were made diabetic via administration of an intraperitoneal injection of streptozotocin (STZ) (S0130, Sigma) at 200 mg/kg in acetate phosphate buffer, pH 4.5 (Solarbio). Animals were considered diabetic when their blood glucose levels exceeded a preestablished value of 15 mmol/l (270 mg/dl) for two consecutive days [19]. One day before transplantation, INS-1 cells were seeded at a density of 5 × 10^5^ cells per well, and a 1 : 1 combination of INS-1 and NG108-15 cells was seeded at a density of 5 × 10^5^ cells per well into 6-well ultra-low attachment, flat-bottomed tissue culture plates (Corning) for 24 hours to form pseudoislets. NG108-15 cells also were seeded at a density of 5 × 10^5^ cells per well to form cell clusters as control. For transplantation, in order to ensure that the number of transplanted beta cells were identical, one-well homotypic PIs or two-well heterotypic PIs were injected subcutaneously into the back of nude mice.

### 2.12. Functional Evaluation of Pseudoislet Grafts

For all transplanted groups, pseudoislet graft function was assessed through nonfasting blood glucose measurements using a portable glucometer (ACCU-CHEK Performa, Roche). Reversal of diabetes was defined as two consecutive readings < 11.1 mmol/l [19], which was maintained until study completion. In addition, glucose tolerance tests were conducted 2 days after diabetes reversal. Animals were fasted overnight before receiving an intraperitoneal glucose bolus (3 g/kg). Blood glucose levels were then monitored at 0, 15, 30, 60, 90, and 120 min after injection, allowing for AUC blood glucose to be calculated and analyzed between the transplanted groups.

### 2.13. HE and Immunohistochemistry Staining

HE and immunohistochemistry staining were performed as described previously [20]. Paraffin-embedded tissues were sequentially sliced at 5 *μ*m thick. Some of the tissue sections were stained with hematoxylin and eosin (HE) (Servicebio), and others were treated with immunohistochemistry staining. For immunohistochemistry staining, paraffin-embedded tissue sections were deparaffinized and hydrated using xylene and graded alcohol to water. Antigen retrieval was performed by incubation of the tissue sections with boiled sodium citrate (Solarbio) buffer (pH 6.0) for 3 min. Endogenous peroxidase activity was quenched with 3% H_2_O_2_. Slides were blocked with 5% BSA (*w*/*v*) (Solarbio) to reduce nonspecific binding and then incubated with insulin (ab7842, Abcam) or *β*-III tubulin (GB11139, Servicebio) primary antibody diluted to a concentration of 1:  100 overnight at 4°C. After incubation with the secondary antibody (Proteintech) for 30 min at room temperature, slides were detected with the DAB Horseradish Peroxidase Color Development Kit (Beyotime) and counterstained with hematoxylin (MX Biotechnologies). Images were taken by a light microscopy.

### 2.14. Statistical Analysis

Data were expressed as mean ± standard deviations (SD). The differences between means and the effects of treatments were analyzed by Student's unpaired *t*-test with two-tailed *p* values and one-way ANOVA followed by Tukey's multiple comparisons test, using GraphPad Prism 6 (GraphPad Software, Inc., USA). A probability (*p*) value < 0.05 was considered to be statistically significant. All experiments were performed at least four times.

## 3. Results

### 3.1. INS-1 Medium Supplemented with HT Is the Ideal Medium for Short-Term Coculture

Hypoxanthine, aminopterin, and thymidine (HAT) were used to culture NG108-15 cells; however, aminopterin in HAT was toxic to INS-1 cells (Figure 2(a)). Due to aminopterin being toxic, it was removed from the coculture media. However, sudden removal of HAT from media caused a significant decrease in viability in NG108-15 cells (Figure 2(b)). Instead, HAT was replaced with HT in the coculture system. In order to ensure *β* cells function properly, INS-1 medium was essential in the coculture system. Because of these reasons, we decided to select INS-1 medium supplemented with HT as the final coculture medium. Compared with the original medium, the coculture medium did not cause a decrease in viability in NG108-15 cells until the third day, and this decrease was less than 10% (Figure 2(c)). This decrease was mainly due to a reduction of glucose content in the medium from 25 mM (DMEM) to 11.1 mM (RPMI 1640) (Figure 2(c)). NG108-15 cells could be passaged successively more than 20 times in the coculture medium and it is enough for the whole experiments to be completed.

### 3.2. Morphological Characterization of Cells in Coculture

**Figure 4:**
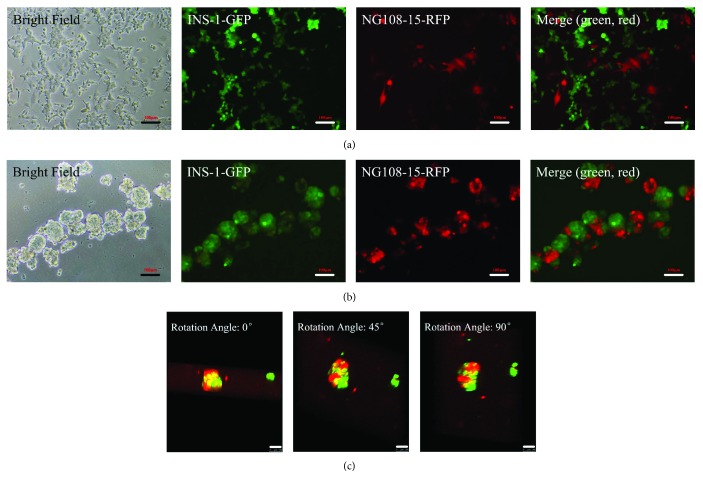
Morphological characterization of cells in coculture. (a) 2D coculture images (10x). (b) Heterotypic pseudoislet images (10x). (c) 3D reconstruction images of heterotypic pseudoislets were taken from different angles (20x). (a, b, and c) Green cells: INS-1 cells and red cells: NG108-15 cells. Scale bars: 100 *μ*m.

Figure 4 shows the morphological characterization of coculture combination of INS-1 and NG108-15 cells in two-dimension and three-dimension seeding conditions. In 2D coculture conditions, NG108-15 cells with red fluorescent protein label and INS-1 cells with green fluorescent protein label both grew as a monolayer on the surface of the well plate (Figure 4(a)). In 3D coculture conditions, NG108-15 cells and INS-1 cells adhered to each other and grew into an islet-like spheroid (pseudoislets) within 24–72 hours of seeding in ultra-low attachment plates (Figure 4(b)). After 72 hours of coculture, heterotypic pseudoislets that formed had a mean diameter of around 200 *μ*m and comprised of approximately 600 cells per pseudoislet.  Figure 4(c) shows the three-dimensional reconstruction images of the heterotypic pseudoislets.

### 3.3. Glucose-Stimulated Insulin Secretion Assay

**Figure 5:**
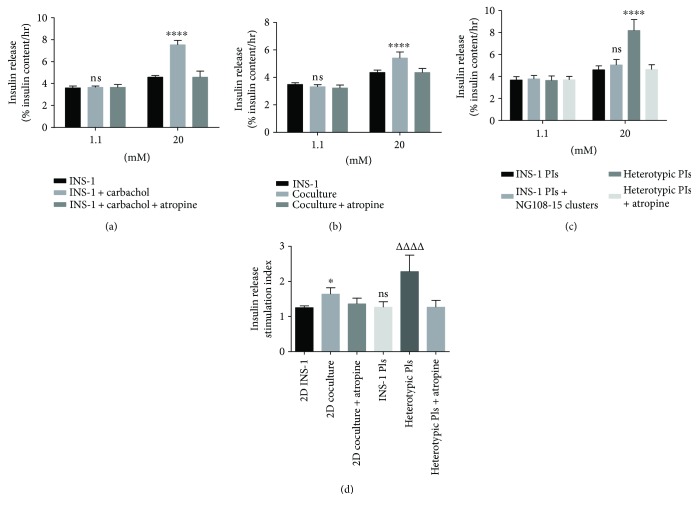
Glucose-stimulated insulin secretion assay. (a) Secretory responses of INS-1 cells with or without carbachol (10 *μ*M) or atropine sulfate (10 *μ*M). ^∗∗∗∗^ *p* < 0.0001 and ns compared to INS-1 group, *n* = 4. (b) Secretory responses of INS-1 cells and 2D coculture cells. ^∗∗∗∗^ *p* < 0.0001 and ns compared to INS-1 group, *n* = 8. (c) Secretory responses of INS-1 PIs and INS-1 PIs + NG108-15 clusters mixture and heterotypic PIs. ^∗∗∗∗^ *p* < 0.0001 and ns compared to INS-1 PI group under 20 mM glucose stimulation, *n* = 8. (d) Stimulation index of all groups. ^∗^ *p* < 0.05 compared to 2D INS-1 group. ^ΔΔΔΔ^ *p* < 0.0001 compared to INS-1 PI groups. ns compared to 2D INS-1 group. (a, b, and c) The data are expressed as percentages of total insulin content. Results are means ± SD of at least four independent experiments.

To confirm that the muscarinic receptor is functional in INS-1 cells, the agonist carbachol and antagonist atropine were used in the glucose-stimulated insulin secretion assay.  Figure 5(a) shows that carbachol (10 *μ*M) can significantly increase the insulin secretion levels of INS-1 cells under high glucose (20 mM) conditions, but carbachol does not affect their insulin secretion levels under low glucose conditions. High glucose induced a greater (1.24-fold increase, *p* < 0.0001) insulin secretion in the 2D coculture group than the INS-1 group (Figure 5(b)). In the 3D culture system, insulin release stimulated by high glucose was even higher (1.78-fold increase, *p* < 0.0001) in the heterotypic PIs group versus the homotypic INS-1 PI group (Figure 5(c)). However, independent NG108-15 clusters could not increase insulin release of INS-1 PIs (Figure 5(c)) suggested the two kinds of cells adhering to each other were necessary for the enhancement effects. Besides, there were no differences in insulin release between the groups at low glucose (1.1 mM) concentrations (Figures 5(b) and 5(c)). When values were presented as stimulation index relative to the 1.1 mM glucose control, the stimulation index in the 2D coculture group and heterotypic PIs group had a 1.29-fold increase (*p* < 0.05) and a 1.81-fold increase (*p* < 0.0001), respectively, when compared to the INS-1 group and homotypic INS-1 PI group (Figure 5(d)). In addition, there was no significant difference in the stimulation index between the 2D INS-1 group and the INS-1 PI group (*p* = 0.98, Figure 5(d)). Additionally, atropine sulfate (10 *μ*M) completely prevented the enhancement effects of NG108-15 (Figures 5(b) and 5(c)). There were no significant differences observed between the coculture and INS-1 groups on insulin release in the presence of atropine sulfate.

### 3.4. E-Cadherin Plays a Key Role in Heterotypic Pseudoislet Formation

**Figure 6:**
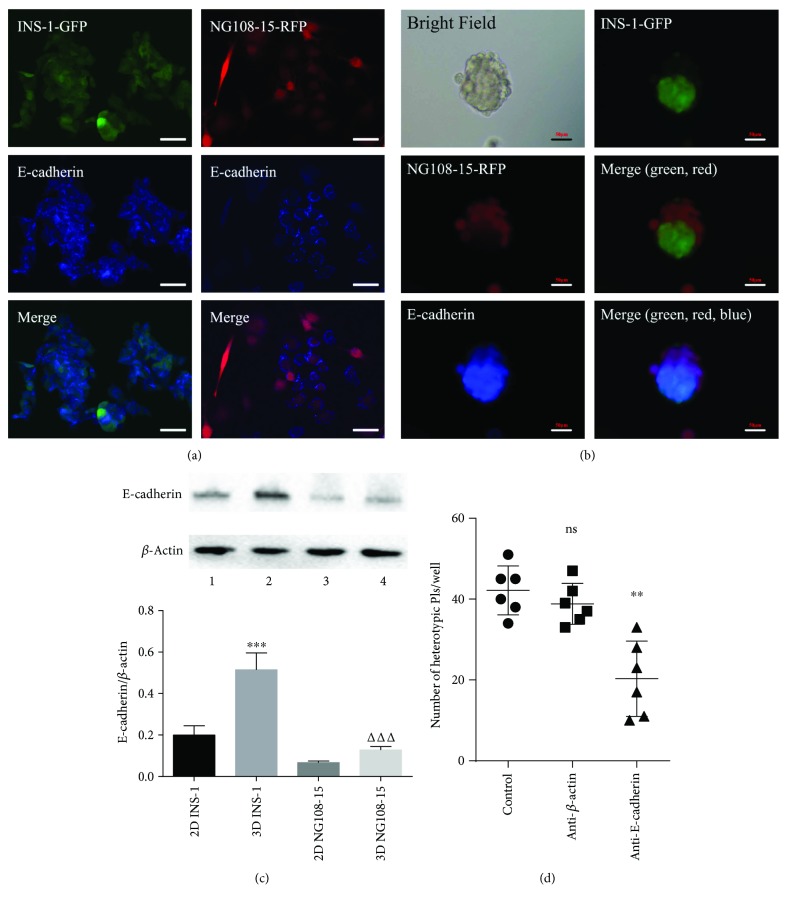
E-cadherin plays a key role in heterotypic pseudoislet formation. (a) E-cadherin was detected in cell climbing slices by indirect immunofluorescence (40x). (b) E-cadherin was detected in heterotypic pseudoislets by indirect immunofluorescence (20x). (a and b) Red: NG108-15 cells, green: INS-1 cells, and blue: E-cadherin. Scale bars: 50 *μ*m. (c) E-cadherin was detected in INS-1 and NG108-15 cells by Western blot. Lane 1: 2D-cultured INS-1 cells, lane 2: 3D-cultured INS-1 cells, lane 3: 2D-cultured NG108-15 cells, and lane 4: 3D-cultured NG108-15 cells. ^∗∗∗^ *p* < 0.001 versus 2D INS-1 group and ^ΔΔΔ^ *p* < 0.001 versus 2D NG108-15 group, *n* = 4. (d) Number of heterotypic pseudoislets formed per well. INS-1 and NG108-15 cells were seeded into 96-well ultra-low attachment plates and maintained in coculture medium (control) with or without E-cadherin antibody or *β* actin antibody for 24 hours. ns versus control group. ^∗∗^ *p* < 0.01 versus anti-*β*-actin group, *n* = 6.

The Ca^2+^-dependent adhesion molecule E-cadherin plays an important role in the formation of pseudoislets [10, 17]. Expression of E-cadherin in INS-1 cells has been reported, but whether NG108-15 cells express E-cadherin have not been reported. Immunofluorescence images demonstrate that E-cadherin is expressed on both INS-1 and NG108-15 cells (Figures 6(a) and 6(b)). Western blot data revealed that cells grown in 3D culture induced the upregulation of E-cadherin expression in both INS-1 and NG108-15 cells (Figure 6(c)). Incubation with anti-E-cadherin antibody inhibited heterotypic pseudoislet formation (*p* < 0.01, Figure 6(d)).

### 3.5. Coculture System Hardly Affects the Proliferation of INS-1 Cells

Carbachol hardly promoted INS-1 cell proliferation by CCK-8 assay (Figure 3(a)). As shown in Figure 3(b), there was no significant increase in DNA synthesis of INS-1 cells after carbachol (10 *μ*M) treatment. In addition, atropine sulfate did not influence the absolute cell number (OD value) of the coculture group (Figure 3(c)). Finally, the OD value histogram suggested there was no significant difference in absolute cell numbers between coculture and independent culture groups (Figure 3(d)).

### 3.6. Subcutaneous Pseudoislet Transplantation Regulates Blood Glucose Levels in STZ Diabetic BALB/c Nu/Nu Mice

**Figure 7:**
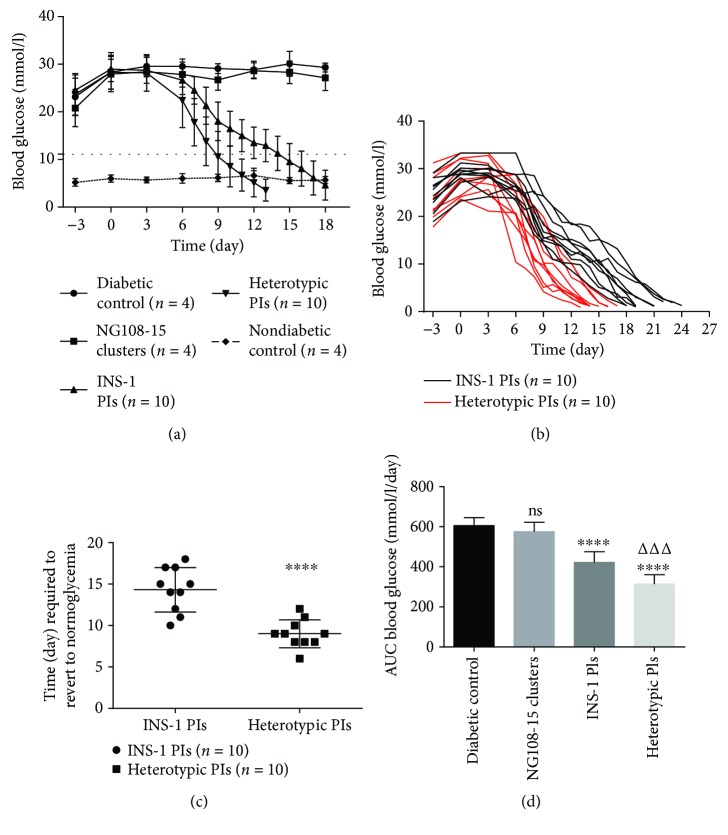
Subcutaneous pseudoislet transplantation regulates blood glucose levels in STZ diabetic BALB/c nu/nu mice. (a) On day 0, pseudoislets or NG108-15 clusters were injected subcutaneously into the back of nude mice. Values are mean ± SD. The dotted straight line represents 11.1 mmol/l. (b) The blood glucose curves of every mice receiving PIs. The end of curves represents the death of mice. (c) Time (day) required to revert to normoglycemic (<11.1 mmol/l) level after subcutaneous PI transplantation. ^∗∗∗∗^ *p* < 0.0001 compared to INS-1 PI group, *n* = 10. (d) Areas under the blood glucose curve expressed as mmol/l/day. ns compared to diabetic control group. ^∗∗∗∗^ *p* < 0.0001 compared to diabetic control group. ^ΔΔΔ^ *p* < 0.001 compared to INS-1 PI group.

STZ caused an increase in mean blood glucose levels from <11 mmol/l to >15 mmol/l within 3 days of administration. Mice receiving heterotypic PIs all reverted to normoglycemic (<11.1 mmol/l) levels within 12 days of transplantation (Figures 7(a) and 7(b)). The mean time required to restore normoglycemia for mice receiving heterotypic PIs was shorter (9 d versus 14.3 d, *p* < 0.0001, Figure 7(c)) than that of INS-1 pseudoislet mice. However, after 12 days, a number of mice receiving PIs began to die due to hypoglycemia (<2.8 mmol/l) (Figure 7(b)). Mice receiving PIs all underwent hypoglycemia and eventually died of hypoglycemia (Figure 7(b)). The heterotypic pseudoislet mice showed a significant decrease (25.6% decreases, *p* < 0.001, Figure 7(d)) in the area under the curve values compared to the homotypic pseudoislet mice.

### 3.7. Subcutaneous Pseudoislet Transplantation on Glucose Tolerance in STZ Diabetic BALB/c Nu/Nu Mice

**Figure 8:**
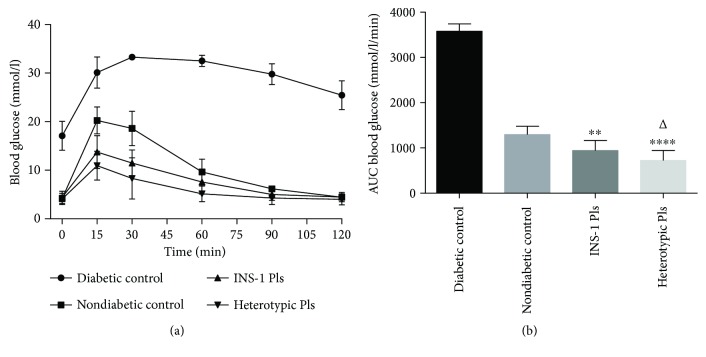
Subcutaneous pseudoislet transplantation on glucose tolerance in STZ diabetic BALB/c nu/nu mice. (a) Two days after diabetes reversal, intraperitoneal glucose tolerance was measured. Values are mean ± SD, *n* = 10. (b) Areas under the blood glucose curve expressed as mmol/l/min. ^∗∗^ *p* < 0.01 and ^∗∗∗∗^ *p* < 0.0001 compared to nondiabetic control group. ^Δ^ *p* < 0.05 compared to INS-1 PI transplantation group.

Following a 12-hour fast and intraperitoneal glucose administration afterwards, the plasma glucose levels in the INS-1 pseudoislet and heterotypic pseudoislet recipient mice were significantly lower (*p* < 0.0001, Figure 8(a)) than those in the diabetic control group for all the time points measured. Additionally, compared to nondiabetic control mice group, the areas under the curve (AUCs) were significantly lower (*p* < 0.01–0.0001, Figure 8(b)) in both pseudoislet transplantation groups. Furthermore, compared to INS-1 pseudoislet recipient mice, the heterotypic pseudoislet recipient mice exhibited lower AUCs (*p* < 0.05, Figure 8(b)). This suggested that heterotypic pseudoislet recipient mice had a greater blood glucose clearance compared to INS-1 pseudoislet recipient mice.

### 3.8. HE and Immunohistochemistry Staining of Heterotypic Pseudoislet Grafts

**Figure 9:**
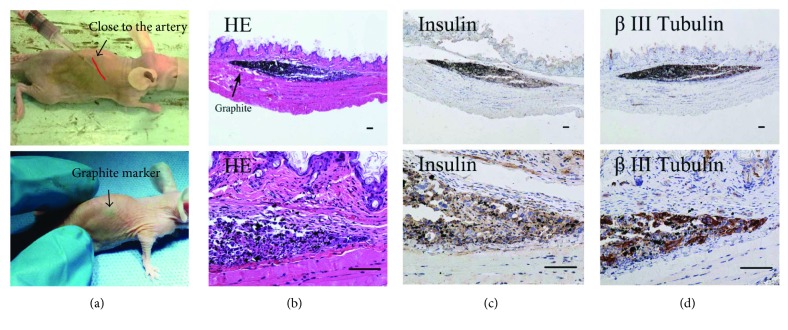
HE and immunohistochemistry staining of heterotypic pseudoislet grafts. (a) Heterotypic PIs mixed with graphite were injected subcutaneously into the back of STZ diabetic nude mice (*n* = 4). The injection site was close to the dorsal artery. Four STZ diabetic mice with graphite marker were only used in histological examination and they were not be used in the previous experiments due to graphite markers. (b) Paraffin sections were stained with hematoxylin and eosin at day 5. (c, d) INS-1 cells and NG108-15 cells were characterized by staining with insulin and *β*-III tubulin in serial sections. (b, c, and d) Scale bars represent 100 *μ*m.

Pseudoislet grafts grew into very small masses under subcutaneous space over the course of the study. These masses were not visible during physical examinations, so we used graphite powder (DK, Tianjin, China) as markers to find the grafts (Figures 9(a)–9(d)). Pseudoislets were gathered into a mass rather than diffusely distributed under subcutaneous space after injection (Figure 9(b)). Subcutaneous heterotypic PI-derived masses showed peroxidase staining for insulin and *β*-III tubulin (Figures 9(c) and 9(d)). The *β*-III tubulin is a specific marker of neurons.

## 4. Discussion

The glucose-stimulated insulin secretion (GSIS) results of 2D coculture confirmed our hypothesis that cholinergic NG108-15 cells enhanced the GSIS capacity of INS-1 *β* cells. However, the enhancement impact of the 2D coculture needed to be further refined to enhance GSIS. We developed a heterotypic 3D pseudoislet model containing two different populations of cells. The two different populations of cells were able to adhere to each other and form an islet-like structure. The GSIS capacity of these heterotypic pseudoislets was enhanced, with insulin release nearly doubling with high glucose (20 mM) stimulation. And the two kinds of cells adhering to each other were necessary for this enhancement effect. We also determined that glucose-stimulated insulin secretion in INS-1 cells was minimally influenced by forming islet-like structures. Furthermore, we found E-cadherin played an important role in the formation of heterotypic pseudoislets which were formed when cells were cultured in ultra-low attachment plates after 24–72 hours. Besides, NG108-15 cells hardly affected the proliferation of INS-1 cells in vitro. Finally, our in vivo work demonstrated that heterotypic pseudoislet transplantation recipient mice reverted to normoglycemic levels faster and had a greater glucose clearance compared to INS-1 pseudo-islets recipient mice.

In the study of heterotypic pseudoislet, hepatocarcinoma cells, glucagon-secreting cells, somatostatin-secreting cells, and GLP-1-secreting cells have been used in these coculture models [15, 21–24]. These cells, to some extent, enhanced the GSIS ability of insulin-secreting cells. Here, we demonstrate for the first time that using cholinergic NG108-15 cells enhanced *β* cell function.

Drs. Kinoshita and Kusamori reported that pseudoislet formation minimally improved glucose-responsive insulin secretion using mouse insulinoma MIN6 and NIT cells [25, 26]. In contrast, Iwasaki et al. reported that MIN6 pseudoislets showed higher glucose-stimulated insulin secretion compared to 2D-cultured MIN6 cells [27]. Our present study demonstrated that glucose-stimulated insulin secretion from INS-1 cells was minimally changed by forming a pseudoislet structure.

The two kinds of cells are tumorigenic, especially NG108-15 cells have very strong tumorigenicity, invasiveness, and migration capability [28, 29]. In addition, the subcutaneous injection of tumorigenic INS-1 cells will cause hypoglycemia of experimental animals [28, 30]. Although subcutaneous transplantation experiments showed the advantages of heterotypic PIs in restoring normoglycemia speed and glucose cleaning capacity, these results were far from being used in clinical practice because of tumorigenicity.

Strategies to establish nontumorigenic *β* cells could provide therapeutic value for the treatment of T1DM. Published studies have shown promising results in developing large numbers of reversibly immortalized cells based on the Cre/loxP system [31, 32]. The use of *β* cells derived from somatic cells or stem cells has also been successfully used to cure diabetes in animal models [33, 34]. However, as mentioned previously, the ability of pure populations of beta cells in vitro and in vivo may not be as beneficial when compared with coculture of relevant supporting cells. Therefore, in order to improve the transplant effect, it may be a good idea to incorporate the relevant partner cells to manufacture artificial islets. Our study demonstrated that cholinergic cells were well suited for coculture. However, generating large numbers of nontumorigenic cholinergic cells have still not been developed. The possibility that the techniques described for the generation of *β* cells could be applied to generate nontumorigenic cholinergic cells. In a future study, we will try to develop a new type of heterotypic PIs which derived from nontumorigenic *β* cells and cholinergic cells. Without tumorigenicity, blood glucose of diabetic animals will be controlled more safely and physiologically after receiving this kind of heterotypic PIs.

In summary, our data demonstrated that coculture with cholinergic NG108-15 cells was able to enhance the GSIS of islet INS-1 cells in 2D and 3D culture conditions, and this effect was mediated via neurotransmitter acetylcholine release. In addition, E-cadherin played an important role in the formation of these heterotypic pseudoislets. Heterotypic pseudoislets derived from INS-1 and NG108-15 cells when transplanted into recipient STZ-treated mice were able to revert to normoglycemic levels faster and attain better glucose clearance when compared to homotypic INS-1 pseudoislet transplantation. Our work provides proof of the concept that combined insulin and cholinergic cell therapy using pseudoislets holds promising therapeutic potential. Additionally, our work contributes important new knowledge to the fields of islet transplantation and *β* cell replacement therapy.
